# Preparation of the graphene-based smart hydrophobic nanocomposite and its application in oil/water separation

**DOI:** 10.1038/s41598-023-46520-2

**Published:** 2023-11-13

**Authors:** Mahsa Alimohammadian, Saeid Azizian, Beheshteh Sohrabi

**Affiliations:** 1 Surface Chemistry Research Laboratory, Faculty of Chemistry, Iran University of Science and Technology, Tehran, Iran; 2 Department of Physical Chemistry, Faculty of Chemistry, Bu-Ali Sina University, Hamedan, Iran

**Keywords:** Materials science, Chemistry, Physical chemistry, Surface chemistry

## Abstract

Designing and synthesizing materials with smart hydrophobicity against an external magnetic field for efficient oil/water separation is of great importance due to the increasing problems caused by oil pollution. Here, the nanocomposites were fabricated based on graphene and different iron oxides exhibit smart hydrophobicity against an external magnetic field and they are in powder form eliminating the requirement for a substrate employing a facile and echo friendly method. The results prove that autoclaving of graphene leads to its ferromagnetic property; then it is attached to iron oxides by magnetic attraction and a nanocomposite is produced. The magnetic property of the resulting nanocomposite is higher than the magnetic property of its individual components. In addition, following nanocomposite formation, its hydrophobicity and surface area also change. FESEM images were taken from the nanocomposites to study their surface morphology, and EDS-MAP analysis to observe the elemental distribution uniformity of the nanocomposites. Also, to measure the surface area and pore size, BET analysis has been performed on pure materials and graphene-black iron oxide nanocomposite (graphene@black iron oxide). The results show that the specific surface area of black iron oxide increases after being composited with graphene dispersed at 5000 rpm. Indeed, graphene forms a composite by binding to iron oxide, and therefore, its specific surface area increases compared to iron oxide and graphene alone. These results show an increase in oil sorption and better separation of oil from water by the prepared nanocomposite. Also, to measure the magnetic properties of pure materials, graphene@black iron oxide, and ferromagnetic graphene at 3000 and 5000 rpm, the Vibrating Sample Magnetometer analysis has been performed. The results have proven that the nanocomposite powder prepared by a simple method obtained from cost-effective and available materials is hydrophobic and becomes more hydrophobic by applying an external magnetic field. Due to the ease with which oil can be readily removed from the nanocomposite by eliminating the external magnetic field, this nanocomposite is an excellent choice for the separation of oil from water.

## Introduction

Aquatic ecosystems have suffered negative consequences as a result of oily wastewaters, petroleum industry spills, and shipping mishaps^1^. These spills can come from a variety of places, including oil rigs, carriers, and undersea pipelines. Studies have suggested a range of ways for cleaning up oil spills, including absorption^2^, dispersants^3^, solidifiers^4^, and controlled burning^5^.  Due to its high efficiency, ease of use, and versatility in combining with other techniques, absorption is one of the most often employed techniques separation and recovery of split oil from water.

Up to now, all methods that have used the phenomenon of hydrophobicity or lipophilicity to separate water from oil have been based on the fabrication of surfaces that have used surface engineering to create roughness and reduce the energy of these surfaces. Unfortunately, the methods used to create roughness and cover the surface with hydrophobic compounds suffer from being costly and time-consuming. These fabrication methods of superhydrophobic surfaces include lithographic methods^6^, layer by layer deposition^7, 8^, plasma etching^9^, chemical vapor deposition (CVD)^10^, template-based extrusion^11^, sol–gel process^12^, electrospinning^13^ self-assembly^14^ and extrusion^15^.

Some of the distinguishing characteristics that oil absorbents should have are high oil sorption capacity, quick oil sorption rate, low density, low cost, and reusability. So far, a wide range of advanced and innovative adsorbents with hydrophobic and oleophilic surfaces have been synthesized to separate and recover oil from water. Fiber sheets^16^, alumina^17^, zeolites^18^, silica^19^, polypropylene^20^, and polystyrene^21^ are a few examples of these absorbents. Some of the limitations of such sorbents that lessen their absorption efficacy are their low sorption capacities, low recovery efficiencies, generation of toxic wastes, and weak buoyant qualities. It is crucial to provide lightweight materials with environmental friendliness, high sorption capacity, and chemical inertness for oil spill response activities. Surface wettability is among the most important factors in the oil–water separation process. One phase may be allowed to permeate while the other is repelled by such regulation of wettability^22^. Thus, superhydrophobic/superhydrophilic membranes and hydrophilic/underwater superhydrophobic membranes are the two main groups of materials or membranes used for water/oil separation. It is water permeable and oil-resistant. For mild oil/water separations, this kind is most effective. Nonetheless, applications for separations involving heavy oil/water are feasible when using materials and membranes with superhydrophobicity. If light oil/water mixes are separated one way alone, it is claimed that light oil molecules will become caught between the water layer and the water-resistant membranes. Therefore, the need for inverse wettability membranes was emphasized as being essential for effective and continuous oil/water separation^22^. Such membranes were made by coating various materials, such as metal oxides, sponges, polymers, metal meshes, and many more.

Solids called composites comprise two or more different constituents or phases on a scale greater than an atom. These materials are distinguishable from each other in macroscopic dimensions^23–26^. The word “composite” is frequently used to refer to materials whose characteristics, such as the elastic modulus, differ considerably from those of homogeneous materials due to the separation of different phases on a scale bigger than atoms. Composites have properties that cannot be represented by any of the ingredients alone. In other words, these components have distinctly dissimilar chemical or physical characteristics, and when combined, they produce a product with attributes distinct from those of the constituent parts. What distinguishes composites from mixtures and solid solutions is that the components of composites remain separate and distinct in the final structure on a macroscopic or microscopic scale. In addition to a vast array of other items, composites are used in pipelines, buildings, roads, bridges, and airplanes. To create composites that are robust, durable, affordable, and light, researchers are figuring out how to increase composites quality^27–30^.

One of the disadvantages of composites is the concentration of stress at the boundary between their construction phases, which can lead to the destruction of the composite in that area. The structure of the composites has been modified by nanotechnology and the fabrication of nanocomposites. A nanocomposite is a composite in which one or more components are less than 100 nm in size. You can find two phases in nanocomposites. Typically, the nanocomposite’s foundation material or matrix, which can be comprised of a polymer, metal, or ceramic, has a crystalline structure in the first phase^31^. The first phase’s strength, electrical conductivity, resistance, magnetic characteristics, etc. are increased in the second phase by dispersing nanometer-scale particles as reinforcements or fillers. Types of nanocomposites include polymer-based nanocomposites, ceramic-based nanocomposites, and metal-based nanocomposites^32–34^. Among nanocomposites, the most attention is paid to polymer-based nanocomposites. For example, Nine et al. developed a method for the producing modified polymer-based nanocomposites by using graphene. These modified nanocomposites have strong mechanical strength and are self-cleaning, therefore can be used to improve superhydrophobic surfaces, which often suffer from mechanical instability after abrasion/scratching, for self-cleaning applications^35^.

Superhydrophobic composite coatings with high mechanical strength, self-cleaning, and other special qualities may be made using graphene, a two-dimensional matrix of carbon atoms in a honeycomb network^36–38^. While a sheet of graphene weighs only 0.0077 g, it can tolerate up to four kilograms^39^. In other words, although it is thin and light, it is extremely strong. It also has a large surface, excellent thermal conductivity, and electricity and many incredible features. Scientists called graphene as “miracle substance” of the century and predicted that it can cause a huge revolution in various industries. Studies show that graphene has a myriad of unprecedented properties, any of which can be used to make extraordinary composites. The addition of graphene can improve bulk materials’ conductivity and strength and assistance in the development of composites of better ability. Additionally, it may be used to make conductive and heat-resistant composites by mixing it with ceramics, polymers, and metals^40–42^.

The magnetic and electrical characteristics of graphene can be enhanced by doping the surface with metal nanoparticles and other organic and inorganic species. The entire nanocomposite can be magnetized if these particles have magnetic characteristics^43, 44^. In addition, magnetized graphene can also be used to prepare magnetic nanocomposites. In our previous work, we were able to magnetize graphene at room temperature with simple methods. Furthermore, we looked into how magnetic field, pressure, and temperature changes affected the ferromagnetic characteristics of graphene^45, 46^. We can also use this graphene, which is magnetized by a simple and cheap method, to prepare smart magnetic nanocomposite.

Graphene-based composites can contain the element iron in its primary form, or core–shell nanoparticles, and or iron oxides. The presence of oxygen-containing groups on the graphene surface, the employment of reducing agents, and other factors affect the fraction of iron nanoforms in various stages of oxidation. Because epoxy, carboxyl, and hydroxyl functional groups are present, magnetic nanoparticles incorporating iron are deposited on graphene oxide sheets^47–49^.

Due to some problems in the absorption of oil and because the methods that have been presented so far all need a substrate for absorption, in this work, it has been tried to use smart superhydrophobic nanocomposite of graphene and iron oxide with high absorption capability and appropriate recyclability.

The membrane’s hydrophobic characteristics, however, might result in the fouling phenomenon, which reduces the membrane’s lifespan. Hence, it is essential to modify the membrane, particularly to increase its hydrophilicity, in order to maximize its functionality. Due to its exceptional thinness and distinct layered structure, graphene and its derivatives have attracted the interest of numerous researchers working on membranes for water treatment during the past several years. These distinctive characteristics make it ideal for oil/water separation by increasing the penetration fluxes and enhancing the hydrophilic and physicochemical qualities^50^.

Some of the biggest difficulties in using graphene derivatives or their composite membranes for oil/water separation are linked to how well the nanoparticles decompose on the membrane surface. More study is still required to determine how the ratio of graphene derivative-based nanoparticles affects the membrane matrix. The greatest performance of membranes requires optimization of the concentration and distribution of the components based on graphene. Moreover, manufacturing the graphene composite membranes on a large scale presents the largest obstacle to a successful use. If these challenges are solved, successful use for water/oil separation and other water treatment applications may be possible. Studies suggest that because of their significant surface area, porous sponges that are three-dimensional (3D) have considerable promise as high-capacity sorbents. Nevertheless, the majority of sponges sold commercially are not extremely hydrophobic, which restricts their use for recovering and cleaning up oil spills^51^. This means that surface modification is necessary to increase the sponges’ hydrophobicity and decrease their water-wettability in order to create the requisite superoleophilic and superhydrophobic oil sorbent. Sponge surfaces are frequently covered with a variety of substances, such as polyaniline, polypyrrole, carbon nanotubes, graphene, nanofibers, and nanocrystals, to boost their superoleophilicity and superhydrophobicity^52^. The typical characteristics of carbon materials are high capacity, high porosity, mechanical integrity, and large pore volume^53^. Much emphasis has been given to the creation of carbon materials with elevated hydrophobicity, oleophilicity, functional qualities, and distinctive thermal properties^54^. The graphene-based sponge (GS), which is both hydrophobic and oleophilic, has a high capacity for absorbing oils and other organic liquids and is very recyclable. These excellent outcomes make the GS an excellent choice for use in oil and water separation^55^. Aerogel and hydrogel materials, as well as foams based on graphene, have all received significant interest in the subject of water/oil separation^56–58^. An efficient way to remove oils from water by employing graphene-coated cotton fibres has also been proposed, which can be used to separate all types of oils and organic solvents from water. The development of useful materials that can successfully separate oil from water seems important^59^.

Despite reports of significant progress and promising applications, the oil/water separation technique still has many hurdles to overcome. More problems are related to the efficiency and productivity of the industry. One of the biggest problems is how graphene and its derivatives are attached to the substrate because they can easily detach from it during use. This is explained by weak van der Waals forces, which are responsible for keeping graphene and its derivatives on the substrate surface. Therefore, it is very important to develop preparation techniques and perhaps use powders that no longer require a substrate.

The last point is related to the industrialization of the water and oil separation processes by graphene and its derivatives. So far, no research has been conducted on industrial scale studies. Instead, all studies have focused solely on small-scale laboratory systems. Scalability tests are required to assess the effectiveness and acceptability of the system at greater flow rates and under more accurate circumstances. However, recent developments in sponges with graphene attached are still weak and poor chemical and mechanical stability. Consequently, in this work, we tried to use magnetized graphene without the need for a substrate.

The most importance novelty of our research is using ferromagnetic graphene in the composite which can help in the preparation of stable nanocomposites. In fact, ferromagnetic graphene can be attracted Iron particles by magnetic attraction. Our group recently searched the preparation of ferromagnetic graphene and reported several methods. This research is the first application of ferromagnetic graphene that is investigated in our group. On another hand, as a great result, these nanocomposites are smart and their interaction with water can change in the presence of magnets.

Seeking an alternative route to improve the separation quality, we used powder instead of the surface, to separate water from oil. For this purpose, the powders of smart superhydrophobic nanocomposites were prepared which respond to a magnetic field. Nanocomposite powders can be easily separated after absorbing oil (petroleum contaminants) by using a magnet. These nanocomposites become more hydrophobic in the presence of magnets; consequently, their ability is increased to absorb oil. As the hydrophobicity of these nanocomposites increases in the presence of a magnetic field, it will be easier to completely separate them from water.

## Materials and methods

### Materials

The ethanol and graphite (99.9%) were bought from Merck Company. The industrial Iron powder (IP) and black iron oxide (BI), red iron oxide (RI) and yellow Iron oxide (YI) were obtained from Sanat Hamoon Company. Sulfuric acid (> 99%), sodium dichromate (98%) and Sodium hydroxide (99%) were purchased from Merck for use in alkaline and chemical engraving of aluminum alloys. Finally, an OES water filtration system (Oklahoma, USA) provided deionized water.

### Methods

Larger graphene flakes were collected by centrifuge and they were removed (Hettich EBA20). Graphite impurities were quantified by ICP spectrometry (ICPS-7000 Shimadzu). A Sigma 700 tensiometer was used to measure the surface tension of an ethanol solution at 298.15 K using the ring technique at atmospheric pressure^60, 61^. Before each measurement, the platinum ring was carefully cleaned and flame-dried. Vibrating sample magnetometer (Kavir, Iran) measurements were made to determine the magnetization of the produced nanocomposite. Raman spectroscopy (TEKSAN, 532 nm) was used to acquire Raman spectra. FESEM (TESCAN) was used to analyse the morphologies. Analyses of the FTIR (8400S) spectrophotometer were carried out by the Shimadzu instrument. Using N2 adsorption at 77 K and a surface area analyzer (PHSCHINA, PHS-1020, and China), the Brunauer–Emmett–Teller (BET) technique was used to determine the specific surface area. The Data Physics OCA15 plus contact angle metre was used to measure the contact angle (CA) on the surface of the samples. Furthermore, the Digimizer programme was used to measure the contact angle.

### Graphene and nanocomposite preparation

**Figure 1:**
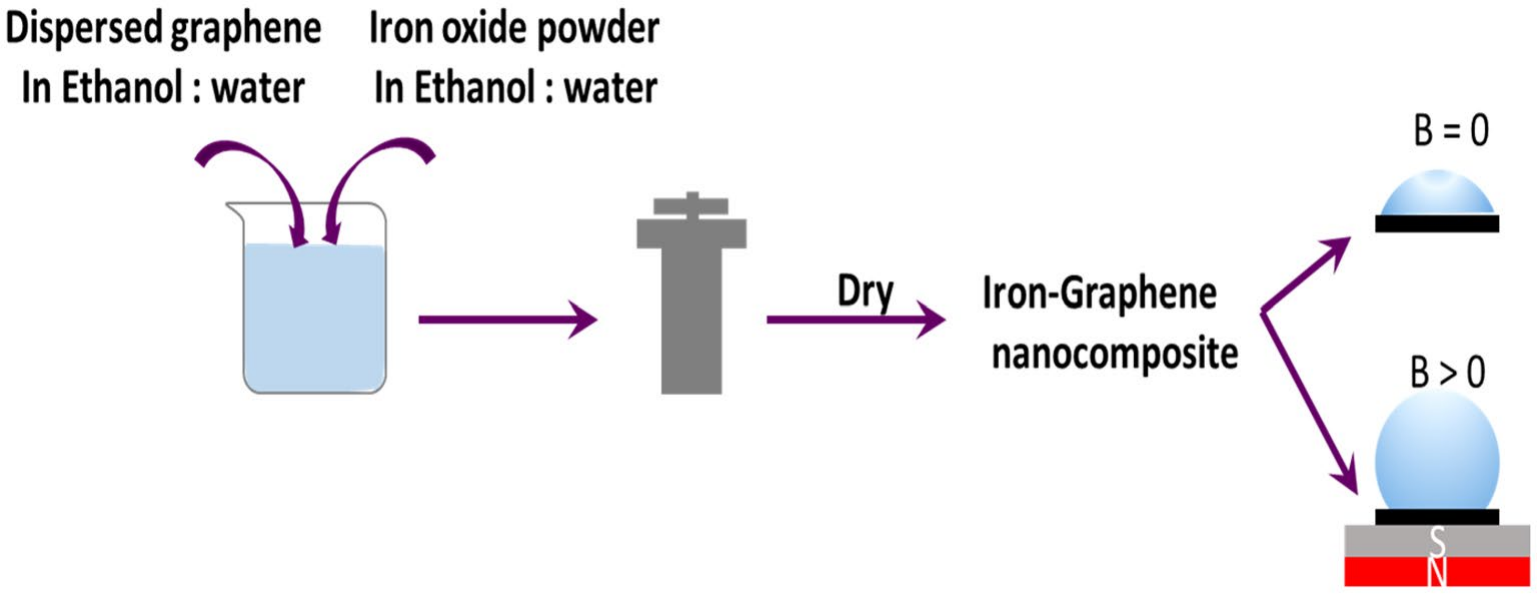
Procedure to generate nanocomposite materials using the hydrothermal process in an autoclave that is heated to 220 °C by means of an oven for 10 h.

By using a process called liquid-phase exfoliation, graphene suspensions were created ^62, 63^. The liquid-phase exfoliation process in an ethanol/water solution was employed to disperse graphene^63^. The key factor for attaining a high yield of graphene dispersion was identified as adjusting the solution’s surface tension in the range of 40–50 mN/m^62^. Various liquids and surfactants are employed to modify surface tension^62–67^. In our previous work, graphene was dispersed using aqueous solutions of two different surfactants as well as their mixtures^66^. In this investigation, a 20:80 ethanol/water ratio was utilised to achieve a surface tension of 45 mN/m. In a mixture of 20:80 ethanol and water, the graphite (5 g/l) was ultrasonically sonicated for 30 min at high power (400 W). The suspensions had been centrifuged at 3000 and 5000 rpm for 10 min to get rid of the bigger flakes. The supernatant from the centrifugation procedure has been decanted and kept in a container in order to employ dispersed graphene in the creation of the nanocomposite. The nanocomposites were prepared in two separate steps with the centrifuged graphene at 3000 and 5000 rpm. According to Table 1, 100 ml of the various solutions containing iron powder and its various oxides were prepared. To prepare these solutions, the mixtures of the deionized water and ethanol (80/20) were prepared, then iron compounds were added to the prepared solutions and sonicated in an ultrasonic bath for 3 min. Nanocomposites were prepared hydrothermally as follows, the prepared graphene at 3000 rpm was mixed by each of the iron solutions prepared above in a ratio of 1:1(Table 1). The mixture was then autoclaved, mixed, and heated for 10 h at 220 °C. The solution (water and ethanol) on top of the precipitation was decanted once the autoclave had cooled, and the precipitate was then dried in a 60 °C oven. The processes for creating a nanocomposite are shown in Fig. 1. Using the same technique, nanocomposites containing graphene at 5000 rpm were also created. The amounts of material (IP, BI, RI and YI) for the preparation of these nanocomposites are presented in Table 2.

**Table 1:** Amounts of iron and its various oxides mixed with graphene 3000 rpm in a ratio of 1:1

Compounds	The amount of material (g)
IP	0.0162
BI	0.0671
RI	0.0463
YI	0.0463

**Table 2:** Amounts of iron and its various oxides mixed with graphene 5000 rpm in a ratio of 1:1

Compounds	The amount of material (g)
IP	0.011
BI	0.0416
RI	0.028
YI	0.028

## Samples preparation

### Preparation of the aluminum sheet

A 30 mm × 30 mm × 0.3 mm aluminium sheet was first degreased in acetone, followed by three minutes of ultrasonic, a rinse in ethanol, and drying. To create a mechanical lock between the coating and the substrate and increase the surface adhesion, the alkali and chemical etching of Al sheet was performed by using sodium hydroxide solution and sulfochromic solution, respectively^68–70^. To this end, 50 ml of 2% solution of sodium hydroxide is poured into a beaker and the aluminum sheet, which has already been washed and dried with acetone in an ultrasonic bath, is immersed in a sodium hydroxide solution, and place the beaker for 2 min in an oil bath that has already been heated to 60 °C. Then, we wash and dry the alkaline aluminum sheet with deionized water. This method causes roughness on the surface of aluminum sheets and oxidation of the aluminum surface.

The sulfochromic solution was prepared according to ASTM D2674 standard. 0.33 g of sodium dichromate powder was poured into a beaker, first, 8.17 ml of deionized water and then 1.83 ml sulfuric acid 98% was added to sodium dichromate. Treated aluminum sheets were immersed in the sulfochromic solution and the beaker was placed in an oil bath that had already reached a temperature of 60 °C for 30 min. Then, the acidified aluminum sheets were taken out and rinsed with deionized water. The oxidized aluminum surface of the alkaline stage was corroded by the acid at this stage, creating micrometer pores in it, which increases the roughness and mechanical locking between the substrate and the composite coating.

#### Coatings on the surface of acidified aluminum sheet

After end of autoclave process, the prepared nanocomposites were dropped on the surface of the chemically etched aluminum sheet and dry at room temperature. Indeed, we prepared a layer of the composites of graphene@iron powder (IPG), graphene@black iron oxide (BIG), graphene@red iron oxide (RIG), graphene@yellow iron oxide (YIG) with the centrifuged graphene at 5000 rpm. In each step, a 10-µl drop was placed on the surface of the aluminum sheet, after drying; the next drop was placed on the previous dried drop until the surface of the sample has been covered with composite. After drying, we measured the contact angle of the composites deposited on the aluminum substrate with deionized water.

In order to better observe the surface changes, FE-SEM images have been prepared from aluminum alloy before etching, after alkaline etching and at the last stage after chemical etching. In the image of the aluminum alloy after alkaline etching (Figure S1), it is observed that micrometer roughness has been created on the surface of the aluminum alloy, which creates more surface area for bonding. After chemical etching (etching with sulfochromic solution), the roughness increased and micrometer holes were formed on the surface of the substrate. During the preparation of the substrate with sulfochromic solution, an aluminum oxide layer is formed on the surface of the substrate, which has micrometer holes. These holes are suitable places for the penetration and attachment of nanocomposites.

### Oil/water separation test

The test was performed qualitatively by pouring deionized water into the beaker, and then 20 µl of oil on the surface of the water was placed. One of the nanocomposites was ground into powder, and the oil absorption by the powder was observed after it was placed on the water surface. The magnet was then brought close to the outer wall of the beaker (from the outside); this was done separately for each of the prepared nanocomposites, and it was looked at how the oil was absorbed by the nanocomposites and how the magnetic field affected the absorption of oil.

## Results and discussion

### The characterization of graphene-based nanocomposites

#### Fourier transform infrared (FT-IR) spectroscopy

On a NICOLET 380 FT-IR spectrometer, Fourier transform-infrared (FT-IR) spectra between 4000 and 400 cm^−1^ have been observed. The produced powder was combined with KBr in a weight-to-weight ratio of 1:4 to create the pellet, which was then compressed. The spectra obtained for the nanocomposites show that graphene isn’t functionalized with iron or its oxides and no new bonds are formed in the graphene structure. In fact, graphene is magnetized in the autoclave^46^, and as a result, it will be connected to ferromagnetic iron compounds through magnetic attraction and form a composite.

**Figure 2:**
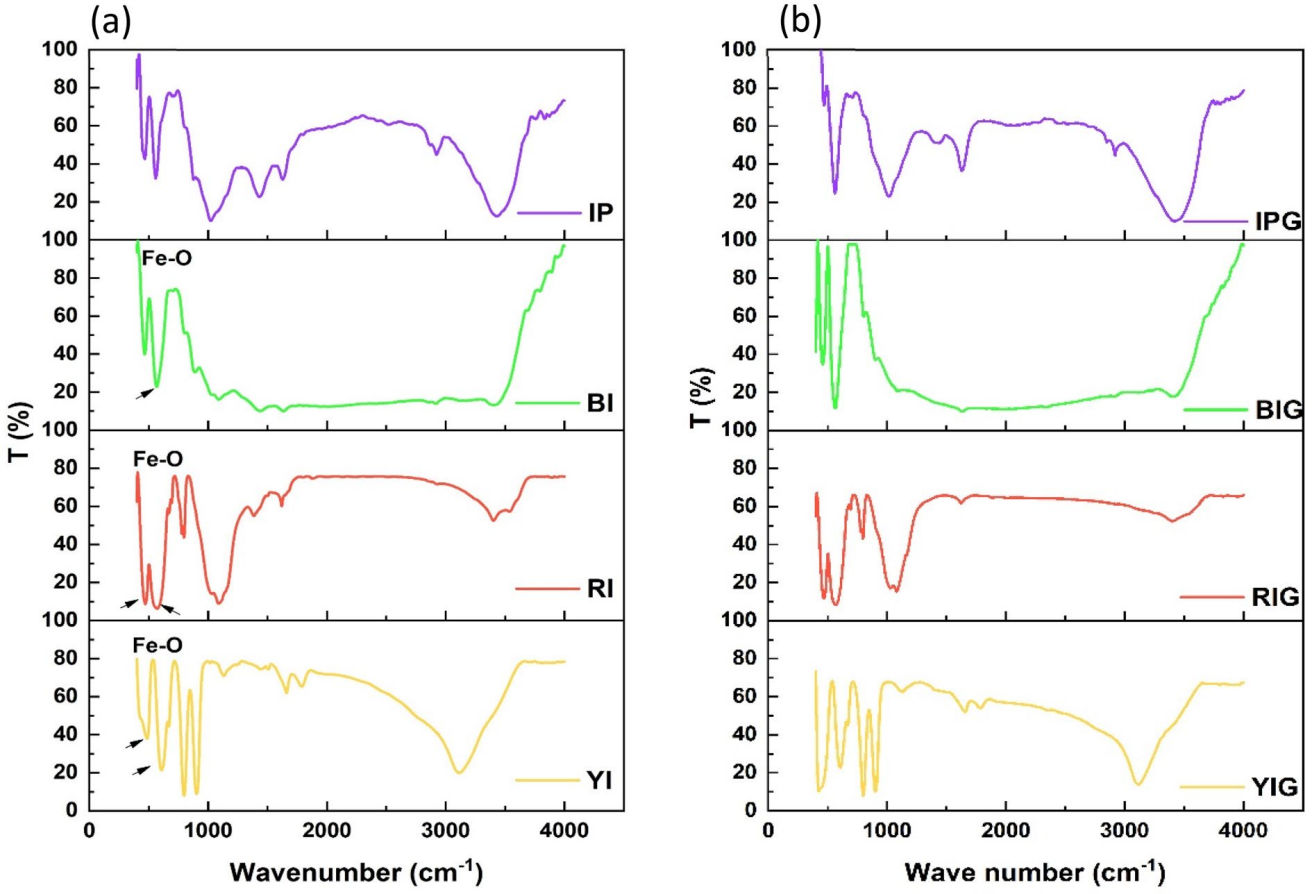
FT-IR spectrum, (**a**) pristine materials. (**b**) Nanocomposites.

FT-IR spectrum of pristine materials (IP, BI, RI and YI) can be seen in Fig. 2. The peak in the area of 566.88 cm^−1^ in BI is connected to the bending vibration of the Fe–O bond^71^. Two peaks at 472.28 and 570.61 cm^−1^ in the FT-IR spectra of RI are associated with the transverse stretching vibrations of Fe–O^72, 73^. Two strong bands at 528 cm^−1^ and 433 cm^−1^ in the FT-IR spectra of hematite nanoparticles are attributed to Fe–O deformation in the Fe_2_O_3_ rhombohedral lattice of hematite. The graphene sheet was incorporated into hematite nanoparticles (Fe_2_O_3_) during the formation of the composite of RIG, as shown by the shift of these bands from 528 to 570.61 cm^−1^ and 433 to 472.28 cm^−1^ in the FT-IR spectrum. The reason for this is the high absorption property of hematite compared to other iron oxides^74^. Additionally, YI provides two peaks in the ranges of 458.156 cm^−1^ and 607.168 cm^−1^ that are connected to the Fe–O bond. A little quantity of water has been absorbed on the samples, as evidenced by the peak in the range of 3300–3500 cm^−1^ in the spectrum of iron and its different oxides, which is connected to the stretching vibration of water. Due to intermolecular hydrogen bonding between FeOOH (YI) and the absorbed water, the surface hydroxyl band in YI is more intense and has moved to 3150 cm^−1^^75^. The structure of FeOOH for YI is clearly shown by the band at 3150 cm^−1^^76^. Eventually, the results obtained from FT-IR analysis confirm the formation of graphene composite with various iron oxides.

#### Raman spectroscopy

**Figure 3:**
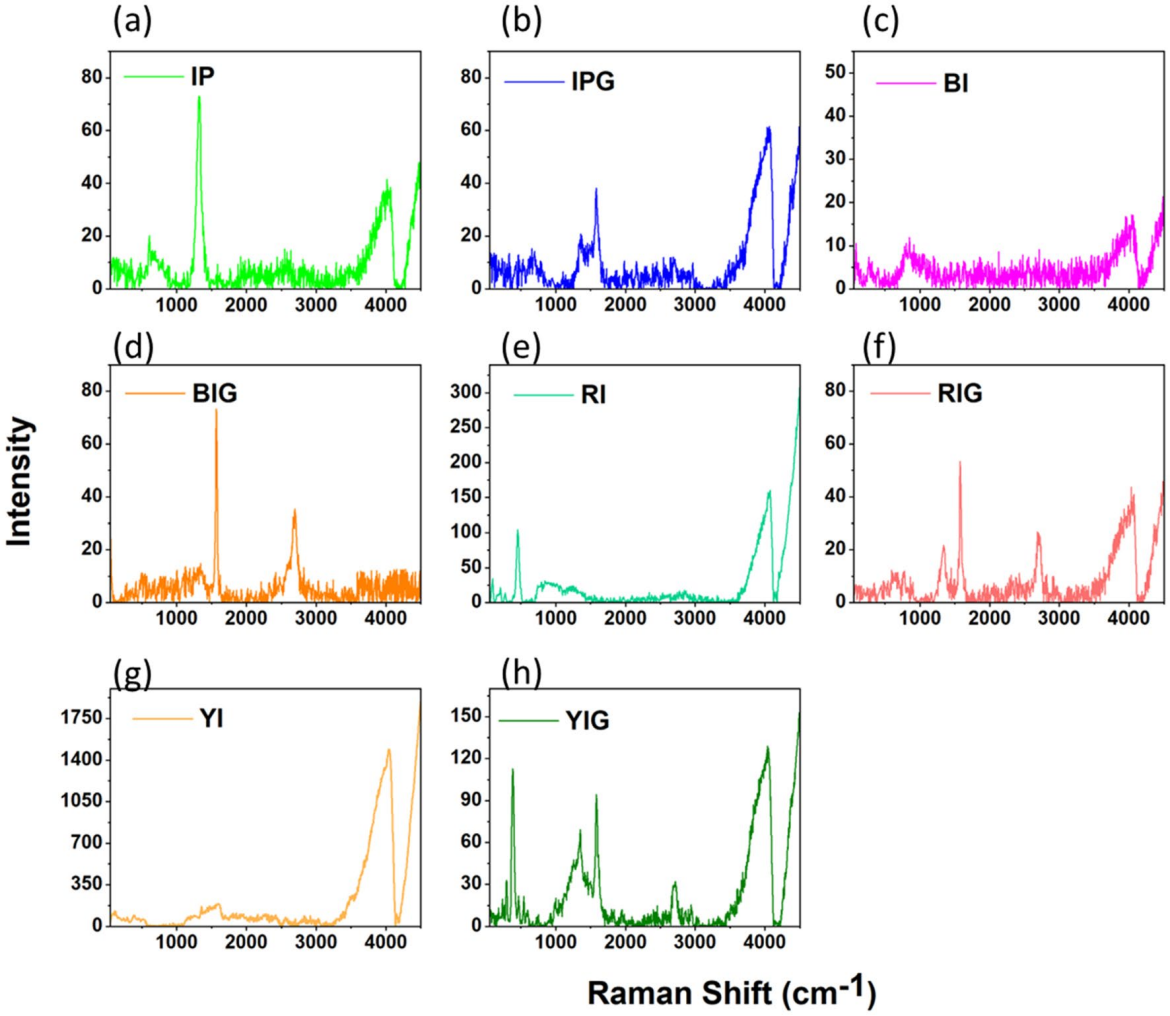
The Raman spectra data. (**a**,**c**,**e**,**g**) Raman scattering of Iron powder (IP), Black iron oxide (BI), Red iron oxide (RI) and Yellow iron oxide (YI), respectively. (**b**,**d**,**f**,**h**), Raman scattering of Iron powder@Graphene (IPG), Black iron oxide@Graphene (BIG), Red iron oxide@Graphene (RIG) and Yellow iron oxide@Graphene (YIG) nanocomposites, respectively. (pure autoclaved graphene at 5000 rpm has been used for preparing of all nanocomposits).

Typically, two Raman and infrared (IR) spectrometers are used to examine the bond vibrations of the manufactured materials. The Raman device picks up non-polar vibrations, while the IR device picks up polar vibrations. Defects in the structure of graphene both before and after the creation of the nanocomposite may be examined using Raman spectroscopy. The most noticeable Raman peak (Fig. 3) is associated with the D band at 1310 cm^−1^, which denotes the existence of defects in the graphene sheet, as well as the peak at 1582 cm^−1^, which is associated with the G band and denotes the vibration of the *sp*^2^ carbon sheet. It is also connected to the two-phonon resonance mechanism that the 2D band seen at 2627 cm^−1^ exists. Any factor that can affect the crystal lattice of graphene and its electronic structure can lead to the change and displacement of all three peaks G, D, and 2D. In other words, due to the exposure of the sample to these factors, the peaks are shifted, broadened or split. Additionally, in most of the spectra (See Fig. 3), structural confusion has appeared in the form of creating new patterns in the spectra. One of the most important factors that can affect the electronic and vibrational structure of graphene is how graphene sheets are assembled. If this accumulation is regular, then the electronic structure and symmetry of graphene will depend on the pattern of placement of the plates. If the accumulation is irregular, there is a possibility of twisting each plate with respect to its upper and lower plates. These modes mostly appear in the area related to interplane vibrations (below 300 cm^−1^)^45, 46^. In this work, autoclave has been used to prepare nanocomposites, since pressure has a major contribution in changing the electronic and vibrational structure of graphene. As a result of this structural disorder, it leads to a disorder in the Raman spectrum and even to the creation of new vibrational modes. In Fig. 3, the D band with lower intensity indicates the presence of fewer defects in the graphene flakes. According to the suggestion of Lotya et al.^64^, there are defects mainly in the edges of the flakes and the basal plan is without defects. The presence of peaks at a wavenumber less than 1000 cm^−1^, which is related to iron oxides, confirms the formation of graphene nanocomposites with iron oxides. The presence of water molecules in the samples may be the cause of the wide peak that was formed in all spectra in the range of 3500–4100 cm^−1^.

#### X-ray Energy Dispersive Spectroscopy (EDS-MAP) analysis and SEM

**Figure 4:**
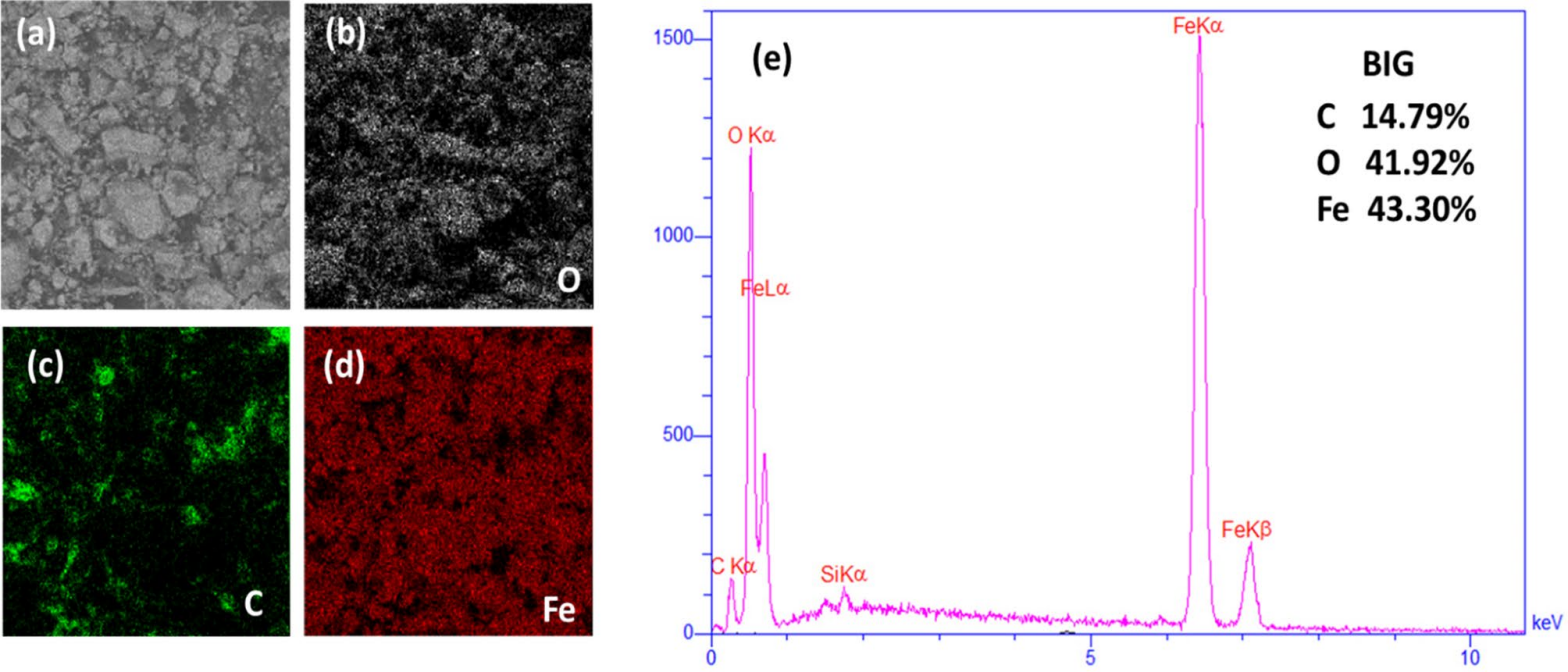
Elemental map and EDS analysis for Black iron oxide graphene nanocomposite: (**a**) SEM (**b**) Oxygen map (**c**) Carbon map (**d**) Iron Map (**e**) EDS spectrum.

In this work, EDS analysis was prepared from pristine IP, BI, RI and YI (Figure S2). EDS-MAP analysis was obtained from IPG, BIG, RIG, and YIG nanocomposites, as well as autoclaved graphene at 5000 rpm. Energy dispersive X-ray spectroscopy (EDS) and scanning electron microscopy (SEM) were used to examine the morphology and chemical composition of the synthesized nanocomposite. Figures S3–S5, and Fig. 4 show the EDS analysis of the nanocomposite of pristine IP and different iron oxides with graphene prepared by centrifugation at 5000 rpm, in which iron nanoparticles and different iron oxides are well combined with graphene layers.

**Figure 5:**
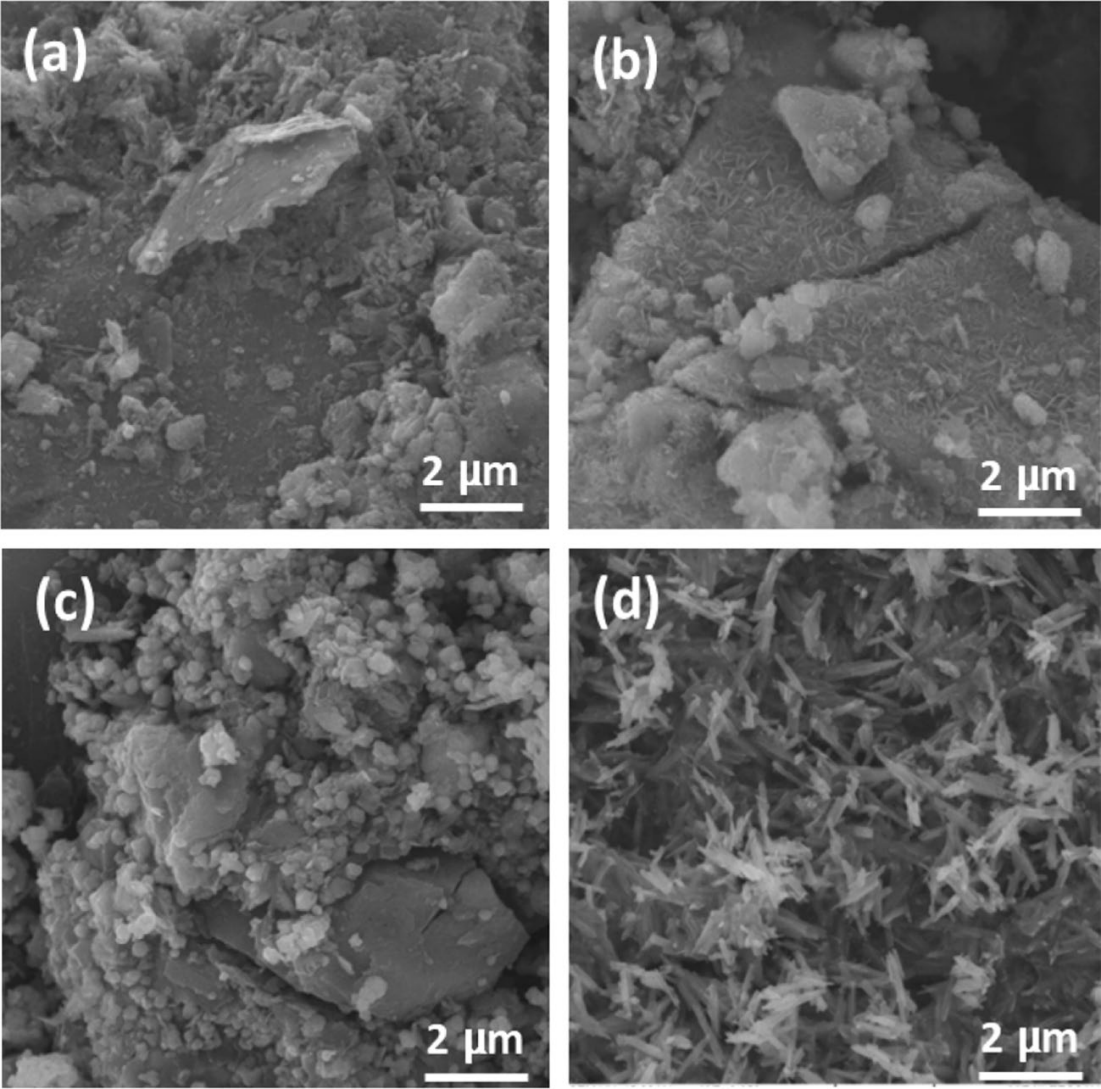
SEM images of nanocomposites: (**a**) IPG (**b**) BIG (**c**) RIG (**d**) YIG.

Iron (Fe) and carbon (C) are the sole elements present in the corresponding IPG EDS spectrum (Figure S3), which proves that a high-purity IPG nanocomposite was successfully synthesised. Also, in Figures S4, S5 and Fig. 4 the EDS spectra show the presence of iron (Fe), oxygen (O) and carbon (C), which indicate the successful synthesis of RIG, YIG and BIG nanocomposites, respectively. Also, the EDS results in Figure S5 show that in the YIG nanocomposite, the percentage of carbon compared to iron and oxygen is lower (about 8%) compared to RIG and YIG. According to the FESEM image in Fig. 5a–d, the YIG structure is needle-shaped (see Fig. 5d); as a result, more iron oxide particles are placed on the surface of the graphene sheet.

The increase in the size of RI and BI particles in the composite prepared with 3000 rpm graphene, which has more layers, leads to an increase in the distance between the particles. The majority of these particles are spherical, as shown in Fig. 5b,c, so as they grow larger due to a reduction in surface area and an increase in steric repulsion, the distance between them grows. As a result, the air trapped between these nanoparticles causes the resulting nanocomposite to become more hydrophobic. As was already mentioned, YIG particles are needle-shaped, according to Fig. 5d. By lowering the centrifuge rpm, increasing the number of needle-shaped graphene layers results in a decrease in spatial hindrance, which in turn reduces hydrophobicity and decreases the distance between the particles.

### Investigation of magnetic properties of graphene-based nanocomposites

In our previous study, we showed that the temperature and pressure inside the autoclave lead to the magnetization of graphene^46^. In addition, iron compounds naturally have magnetic properties. Therefore, the combination of ferromagnetic graphene and magnetic irons can produce a stable composite in which all particles are bound together by magnetic attraction. In this study, the magnetic properties of all components are investigated by the vibrating sample magnetometer (VSM) at ambient temperature, in the field range of − 15 kOe < H <  + 15 kOe.

#### Iron compounds magnetization

**Figure 6:**
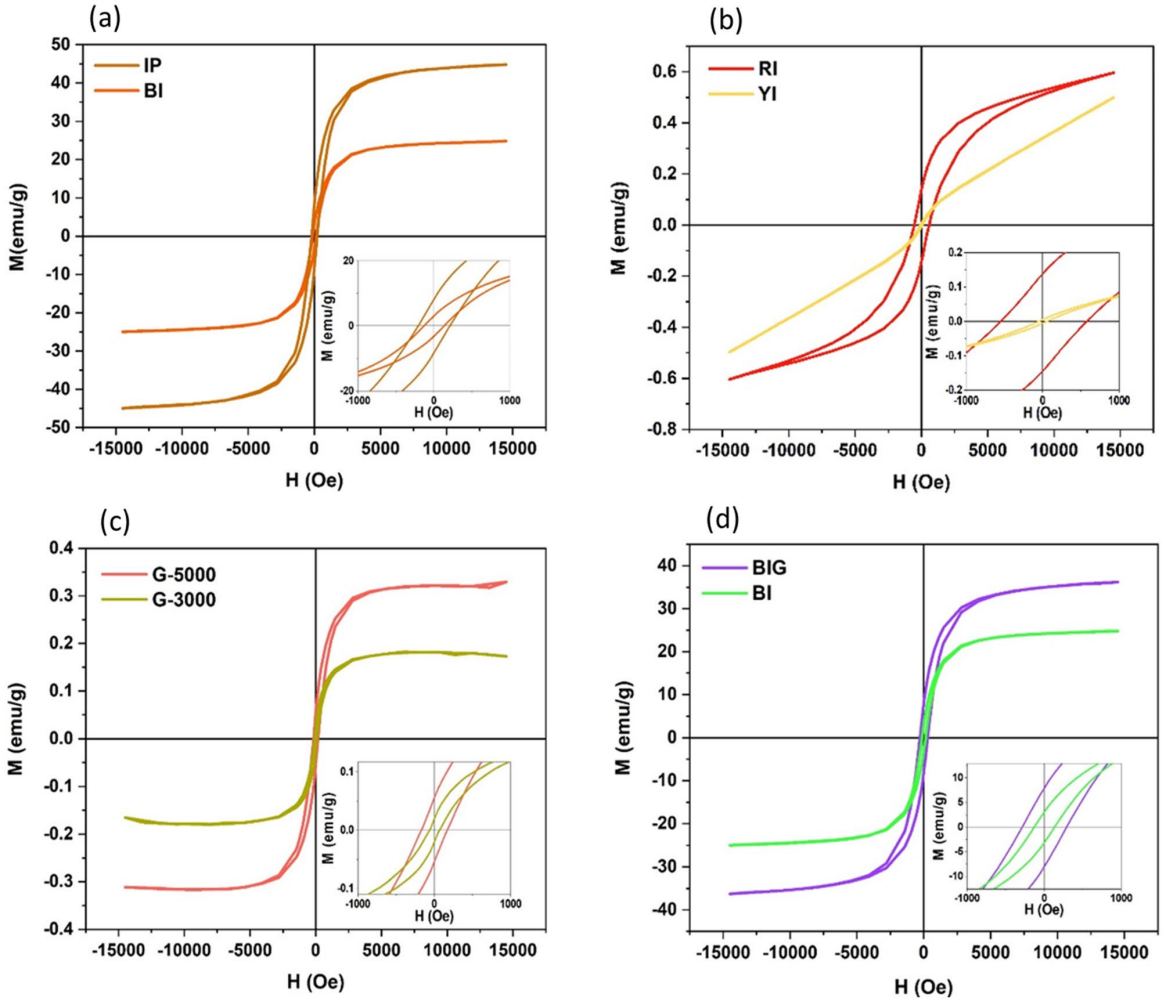
The vibrating sample magnetometer (VSM) for (**a**) Pristine IP and BI (**b**) Pristine RI & YI (**c**) The autoclaved graphene 3000 rpm and 5000 rpm (**d**) BIG & BI. Inset has been zoomed in − 1000 to 1000 Oe, the data was summarized in Table S1.

As shown in Fig. 6a, IP and BI have strong ferromagnetic properties with the highest saturation magnetization (Ms ~ 45 and 25 emu/g), respectively. By contrast, based on Fig. 6b, RI has weak ferromagnetic properties (Ms ~ 0.6 emu/g) and YI shows paramagnetic properties which are different in all iron compounds.

#### Graphene magnetization

Previously, we used graphene flakes prepared by a 3000 rpm centrifuge to magnetize graphene by varying the temperature and pressure inside an autoclave^46^. In this study, the centrifuge at 5000 rpm is used for preparing graphene which its magnetization is more than 3000 rpm sample (Fig. 6c). The probable reason for this observation is the number of layers. Noticeably, the electronic system of thin flakes can be changed more easily than thick flakes whose electrons are involved in van der Waals interactions between layers. Generally, a higher centrifuge speed can remove bigger flakes in lateral size and thickness. Therefore, the 5000 rpm flakes have a lower number of layers in comparison with the 3000 rpm flakes.

#### Nanocomposite magnetization

As shown in Fig. 6d, the combination of ferromagnetic graphene (5000 rpm) and BI can increase the total magnetization in comparison with pristine BI. This behavior has an important role in the water cleaning process. In fact, after the absorption of oils by the composites, the composites can quickly collect with the magnet^45, 46^.

### Magnetic smart hydrophobicity (MSH)

The contact angles of pure materials and nanocomposites in the absence and presence of the magnetic field were measured and compared in order to explore the smart response of synthesized nanocomposites to the magnetic field. The contact angle of pure materials and nanocomposites in the form of powder, tablets, and deposited on the etched Al substrate was measured in the presence and absence of a magnet to demonstrate that a powder with hydrophobic properties with respect to the external magnetic field can be used in the separation of oil from water.

#### MSH of pristine compounds

##### **Iron compounds**

**Figure 7:**
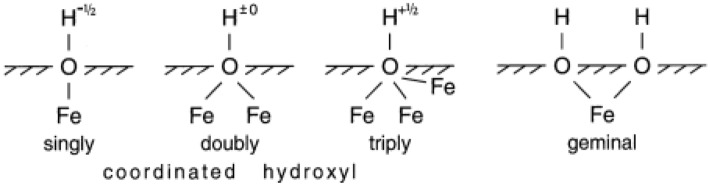
Surface hydroxyl groups on iron oxides and iron powder that are singly, twice, three times, and geminally coordinated^72^.

**Figure 8:**
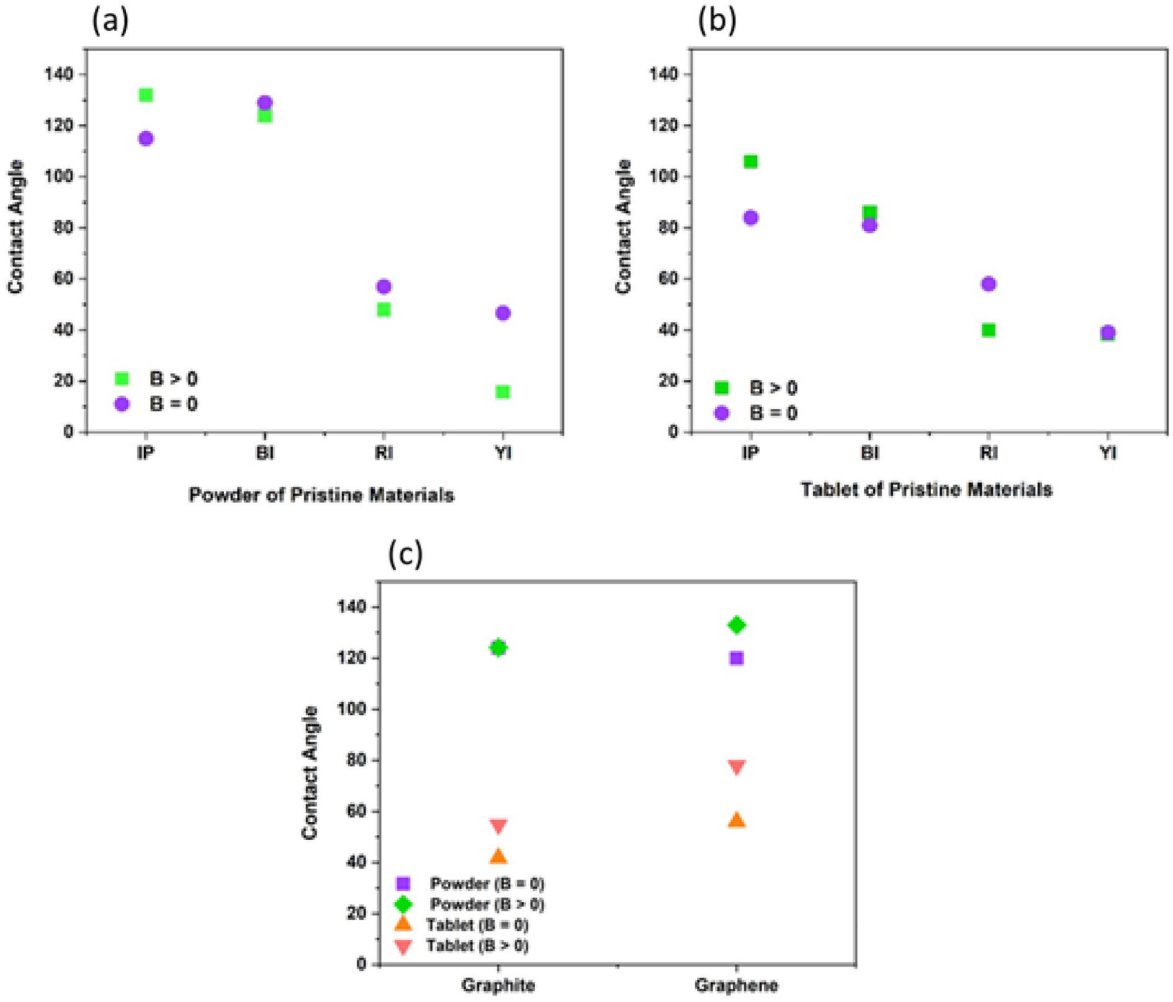
The contact angle of pristine materials (**a**) Powder (**b**) Tablet (**c**) The contact angle of graphite and graphene (rpm) both when a magnetic field is present and absent.

The contact angle of all iron compounds is determined and the results show that IP and BI are hydrophobic and RI and YI are hydrophilic. Following thorough research, it was discovered that iron oxides often consist of tiny crystals with sizes ranging from a few tens of nanometers to a few microns. Because of this, these crystals have a large surface-to-volume ratio, and a large portion of their atoms are on their surface. These crystals have a high surface free energy (in the order of tens of kJ mol^−1^), and reactions at the interface between these iron oxides (Hematite, a red iron oxide, Magnetite, a black iron oxide, and Geotite, a yellow iron oxide) with the solution affect their crystallisation and dissolution, stability, and rheology, as well as their interaction with other species. Surface Fe atoms are Lewis acids and react with Lewis bases because iron oxides have empty atomic orbitals. They consequently coordinate with water molecules or hydroxyl ions in aquatic settings where Fe and these ions share ion–electron pairs. Normally, the water molecules split apart during adsorption, covering the surface with hydroxyl groups that are coupled to the underlying Fe atoms. Iron oxides undergo a quick process called hydroxylation that takes minutes or hours to complete. It is followed by further water molecule adsorption to the surface OH groups through hydrogen bonding. Adsorbed water layers have different characteristics from bulk water; an “ice-like” structure has been proposed. Figure 7 shows that crystallographic investigations demonstrate that surface hydroxyl groups can align with one, two, or three underlying iron atoms^72^. Iron oxide surfaces can have three different types of OH groups with charges of − 0.5, 0, and + 0.5 if a charge of + 0.5 is taken into account for each Fe–O bond (under the assumption of six-fold coordination). Both the crystal structure and the rate at which certain crystal faces form determine the density of these groups. Consequently, it differs slightly from oxide to oxide and is also impacted by crystal shape. The geminal group, which consists of one iron atom and two OH groups, is the fourth kind of group^72^. For this reason, the behaviors of iron compounds are different in terms of hydrophilicity and hydrophobicity. IP and BI are hydrophobic, which shows the hydroxyl group on their surface is coordinated with two iron atoms or is geminal type. YI and FI are also hydrophilic, which indicates coordination with one or three Fe atoms^77^. Additionally, all substances exhibit magnetically smart behaviours, meaning that when magnetic fields are present, their contact angles vary. In the presence and absence of magnets, respectively, nearly BI and YI in powder form had the largest and lowest contact angles, as illustrated in Fig. 8a. When magnets are present or absent in the tablet form, IP has the maximum contact angle, and YI has the lowest contact angle (Fig. 8b). As can be observed from Fig. 8a,b, the presence of magnets increased the contact angle of IP in the form of powder and IP and BI tablets, or, to put it another way, enhanced the hydrophobicity of these two substances. Conversely, the contact angles of RI, BI and YI in the form of powder and RI and YI tablets are reduced in the presence of magnet, i.e., their hydrophilicity is increased. Also, the magnetic sensitivity of IP is more than BI. In addition, both IP and BI in the form of powder are more hydrophobic than tablets. In fact, owing to the reduction of roughness in the tablet form, the contact angles have decreased.

In powder form, the highest magnetic sensitivity in pure material is related to YI and the lowest sensitivity is related to BI. In the case of tablets, the highest magnetic sensitivity is related to IP and the lowest to YI. The contact angle of pristine powder and tablet compounds under the influence of magnetic field varies from 5° to 30.8° and from 0.5° to 22°, respectively.

**Figure 9:**
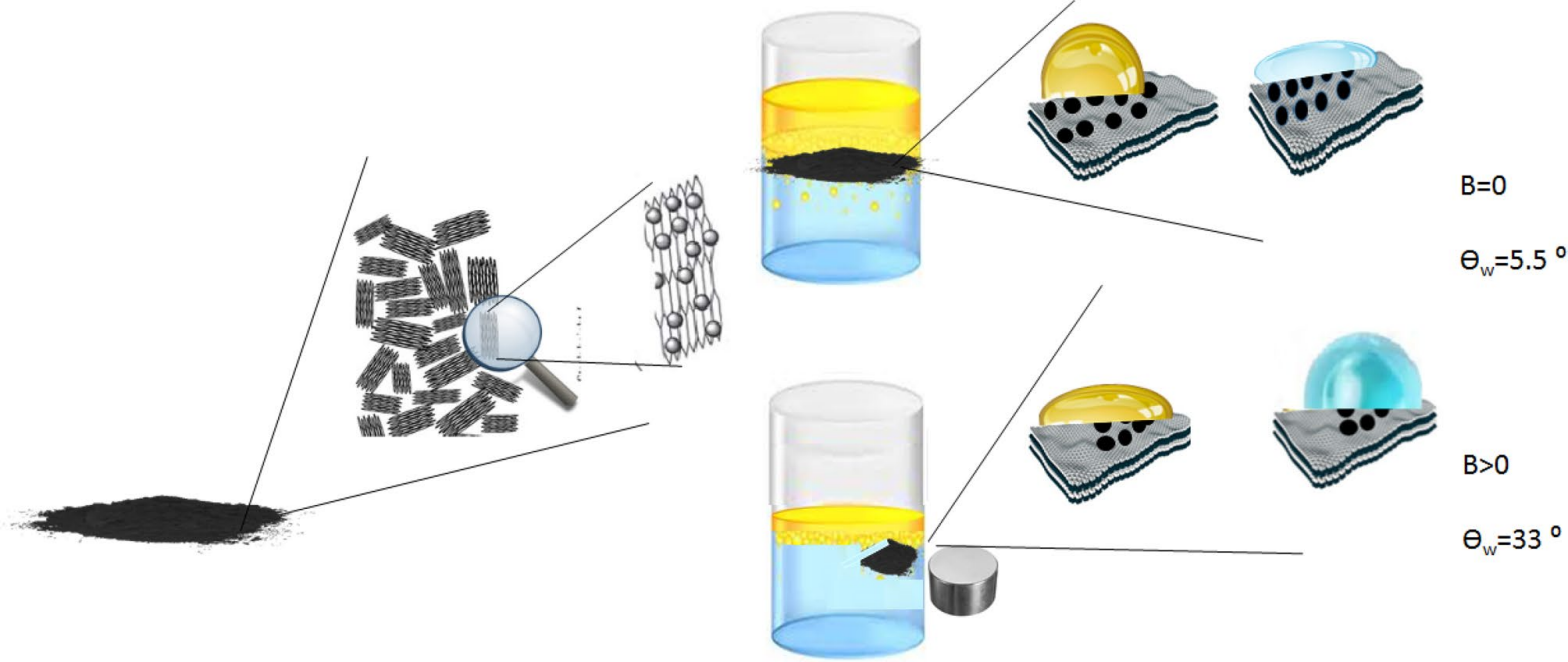
Scheme of the oil/water mixture on the surface of the magnetized nanocomposite powder in the absence and presence of a magnetic field.

The oleophilicity of the composite is modified by applied magnetic fields, although it is not yet clear how. It is clear that the magnetic field must interact with graphene and the ferromagnetic nanoparticles included within the graphene sheets in order to alter the properties of the composite, as the magnetic field has no impact on the interfacial tension of the oil and water. The findings demonstrate how the magnetic field modifies the structure of iron oxide nanoparticles placed on graphene sheets, hence altering the surface’s actual roughness. Figure 9 qualitatively illustrates this suggested method. When a magnetic field is not present, the majority of the nanoparticles are initially dispersed practically on the surface of the graphene sheets, which is consistent with the SEM picture in Fig. 5b. The droplet can rest on top of the nanoparticles with air trapped below because there are significant distances between them (i.e., a Cassie-Baxter model). The nanoparticles, on the other hand, suffer a torque in the direction of the applied field when the magnetic field is applied^78^. The nanoparticles bend in the direction of the field as a result of this torque. Due to the oleophilicity of the graphene sheets, this conformational shift increases the quantity of oleophilic surface that is exposed to the oil droplet, hence enhancing the surface’s oleophilicity. The modified Cassie model, the Wenzel model, or the more complicated “mixed” model may all apply to the oleophilic condition when a magnetic field is present^79–82^. To identify model types when a magnetic field is present, more tests are required.

##### **Graphite and Graphene**

According to Fig. 8c, the contact angle of graphite in powder form is the same in the presence and absence of a magnet. But after turning it into a tablet, its contact angle in the presence of a magnet is greater than its contact angle in the absence of a magnet. It means that it becomes smart and changes its response to the magnetic field. Significantly, it may become magnetized under the pressure applied in the tablet formation process. According to our previous research experience, pressure can induce magnetic behavior in graphene^46^. Thus, under the pressure, graphite can probably be magnetized too and show magnetic smart behavior. The contact angle of autoclaved graphene increases in both the tablet and powder forms in the presence of a magnet. The contact angle of autoclaved graphene in the tablet form is greater than the graphite in the presence or absence of a magnet. And its contact angle in the form of powder in the presence of a magnet is greater than that of graphite in the same conditions. Graphene is more hydrophobic than graphite, because on the one hand, graphene is obtained from exfoliation of graphite, therefore fewer layers and more flexibility lead to wrinkling, rippling and crumpling of graphene and its hydrophobicity increases. On the other hand, the use of an autoclave leads to the change of the graphene’s electrical structure and as a result it’s magnetization. For this reason, if an external magnetic field is present, the wrinkling of the graphene surface increases and its hydrophobicity increases.

#### MSH of nanocomposites

**Figure 10:**
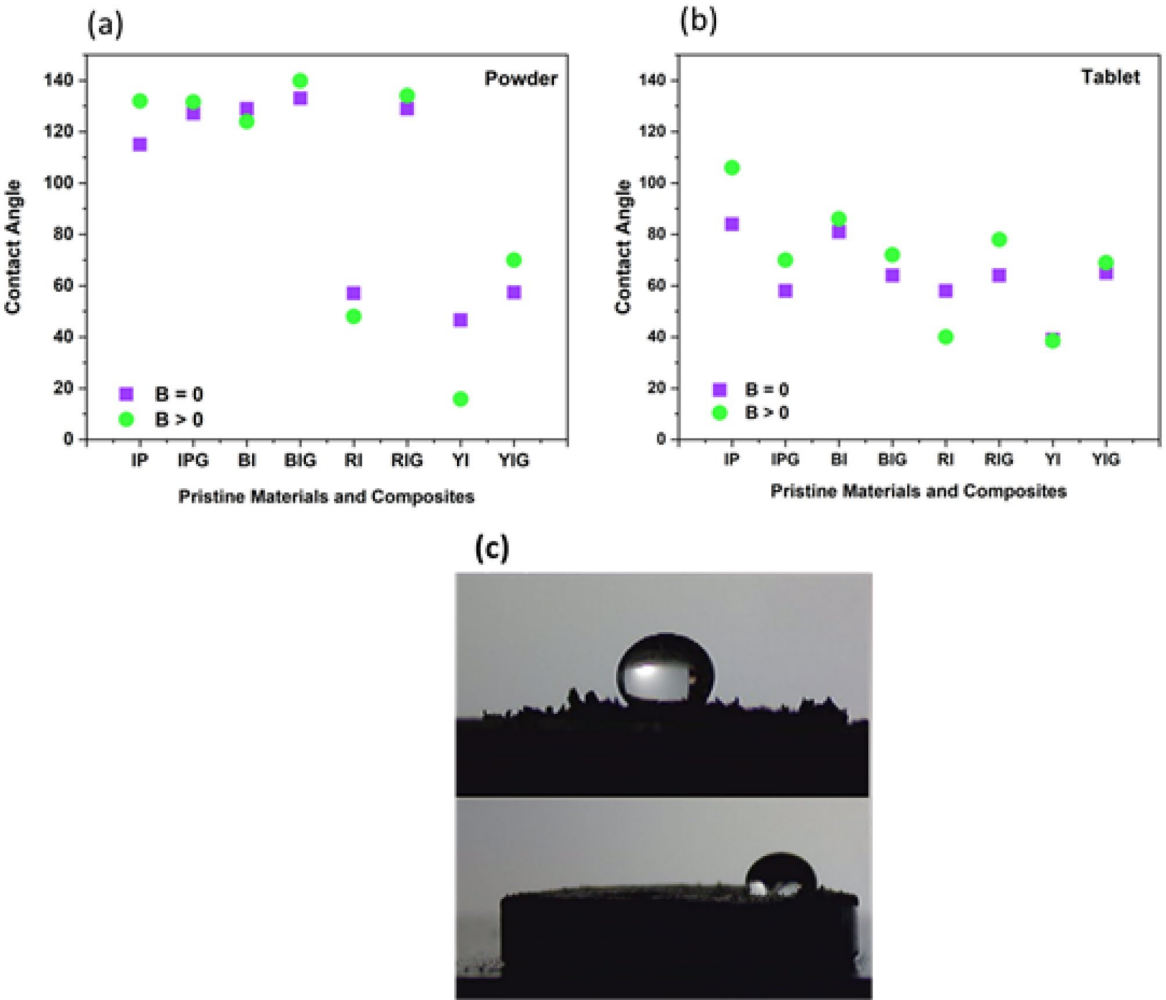
Contact angle of pristine and nanocomposite both with and without a magnetic field (**a**) in the powder form and (**b**) in the tablet form (**c**) Contact angle image of powder and tablet form of BIG nanocomposite.

Figure 10 shows the contact angle of pristine and nanocomposite in the present and absence of magnetic field in the powder form (10-a) and in the tablet form (10-b). As can be seen, in the powder form compositing graphene with various iron oxides leads to increasing the contact angle and thus made the material more hydrophobic. The reason for increasing the contact angle after compositing is probably due to the changes in the structure of pure materials following the use of the hydrothermal method to prepare nanocomposites, which increases their magnetic properties. Except for IP and BI, the contact angle of pure materials in the form of tablet is greater than that of nanocomposite tablets. According to the contact angle data, compressing IP and BI to form a tablet has little impact on its roughness, but due to the mixing of graphene during the preparation of the nanocomposite, the compression of IP and BI to make a tablet has likely decreased the surface roughness. This has reduced the contact angle of the IP and BI in the form of a tablet after composite compared to its pristine form. The results show that in the powder form, BIG nanocomposite has the highest contact angle (Fig. 10c) and YIG nanocomposite has the lowest contact angle. Also, red iron oxide has a huge change in nanocomposite powder form and becomes more hydrophobic than its pristine form in the presence of a magnet. Hence, compositing graphene with iron oxides leads to a smart response of nanocomposites to the magnetic field and changes their hydrophobicity both with and without a magnetic field. In addition, the magnetic sensitivity of nanocomposite is decreased in most cases.

#### Effect of nanocomposite physical form on MSH

**Figure 11:**
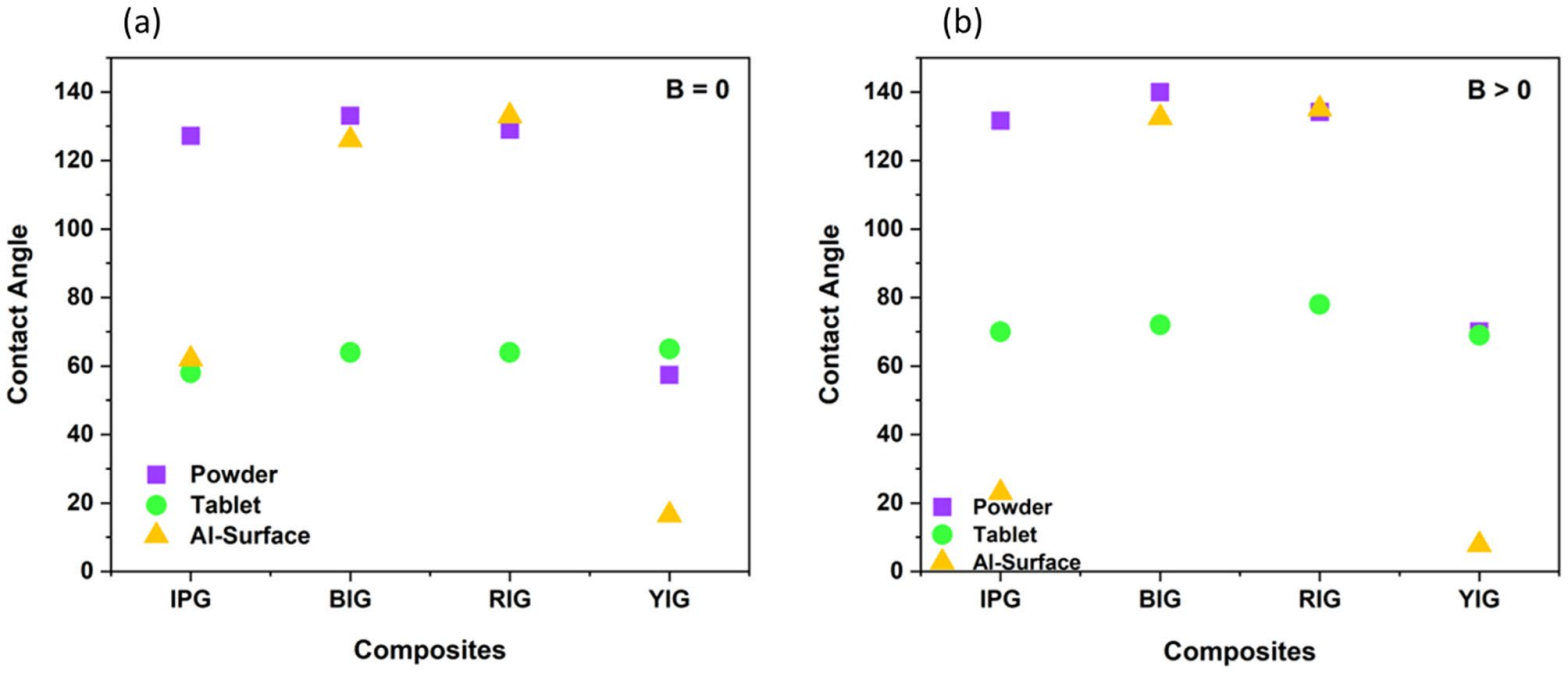
In the absence of a magnet (B = 0) and in the presence of a magnet (B > 0), respectively, the contact angle of powder, tablet, and substrate nanocomposites is shown in Fig. 1. B = 0 in the absence of a magnet, and B > 0 when a magnet is present.

In most cases, the compaction of nanocomposites and their conversion into tablet reduces their contact angle (Fig. 10). On the one hand, smoothing the surface of the material and reducing the roughness caused by compression to prepare tablet reduces the contact angle. On the other hand, reducing the magnetic properties of materials due to their compression during tablet preparation can reduce the contact angle. In all forms of tablet, powder, and substrate, almost all nanocomposites are smart in the presence of magnets. As Fig. 10 shows, the contact angle of some nanocomposites increases in the presence of a magnet and decreases for others. n nanocomposites, the largest contact angle is associated with the powder form of BIG nanocomposite, and the lowest contact angle is associated with YIG nanocomposite deposited on the aluminium substrate, whether a magnetic field is present or not, as shown in Fig. 11a,b. In the nanocomposites deposited on the aluminum substrate, due to the increase in the roughness of the substrate, mechanical locking and strong connection between the substrate and the nanocomposite during the etching stage, the contact angle decreases. The contact angle of IPG and YIG nanocomposites decreases in the presence of a magnet. In other words, these nanocomposites become more hydrophilic in the presence of a magnet. IPG nanocomposite on the substrate is hydrophilic and its contact angle is reduced relative to the powder and tablet forms. The best response of nanocomposites to the magnetic field in the three powder, tablet and substrate states is related to the powder nanocomposite.

#### The effect of graphene layers’ number on the nanocomposites contact angle

Investigating the change in the physicochemical properties of graphene by changing the number of its layers is one of the most important recent research works. Studies have shown that the shape of the metal oxide composited with graphene is influenced by the number of graphene layers^83, 84^. The results of contact angle, X-ray diffraction pattern, Raman, and field emission scanning electron microscopy (FESEM) measurements demonstrate that an increase in the number of graphene layers has a significant impact on the morphology of different iron oxide particles, including their size and the distance between them when they form a composite with graphene. Multilayer graphene also produces particles with larger sizes and distances^84^.

**Figure 12:**
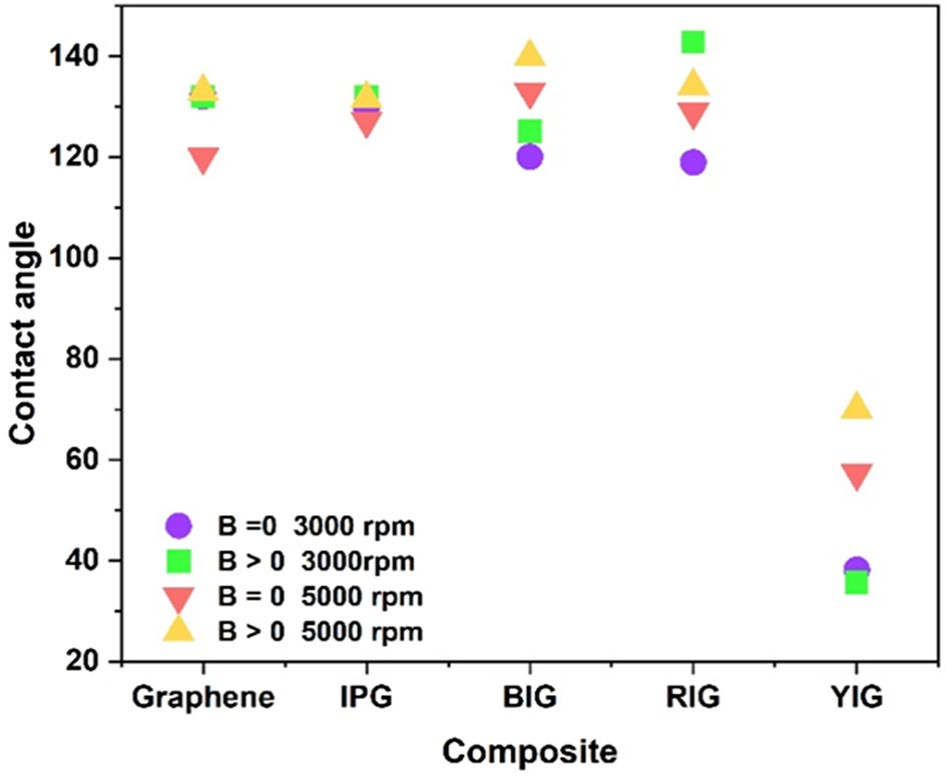
Contact angle of graphene and the nanocomposite of graphene with the various iron oxides 3000 and 5000 rpm in the presence and absence of a magnet.

Figure 12 shows the contact angle of the pure autoclaved graphene at 3000 and 5000 rpm and prepared graphene nanocomposites with pristine iron powder and different iron oxides. The structure of graphene is flexible and it becomes more flexible by reducing the number of layers. For this reason, in the hydrothermal method, the structure of 5000 rpm graphene, which has fewer layers compared to 3000 rpm graphene, is more affected. Since the magnetic field has a greater impact on it as a result of the contact angle data, it behaves smarter when the field is applied. The results presented in Fig. 11 show that the nanocomposites prepared with graphene behave smartly under the influence of a magnetic field at 3000 and 5000 rpm. Of course, the extent of this behavior is not the same in the prepared nanocomposites. The 5000 rpm autoclaved graphene has less number of layers; consequently, its magnetization is higher (Fig. 6c), as a result, it has a better response to the magnetic field than 3000 rpm autoclaved graphene. Therefore, it is more sensitive than graphene 3000 rpm (Fig. 12). However, our results showed that the highest contact angle and smartness in the presence of a magnet are related to red iron oxide graphene nanocomposite prepared with graphene at 3000 rpm. Also, the results of Fig. 11 show that all nanocomposites prepared with graphene become more hydrophobic at 3000 rpm in the presence of a magnet, except the yellow iron oxide graphene nanocomposite. In addition, at 5000 rpm, all nanocomposites became more hydrophobic in the presence of a magnet.

### The study of oil absorption by graphene and various iron oxides nanocomposites powder

#### Determining the specific surface area of nanocomposites

To determine the specific surface area and pore size, which are two effective parameters in oil absorption, BET analysis was performed for BIG nanocomposite, in addition to the pure materials (IP, BI, RI and YI). Thus, the efficiency of this nanocomposite for oil absorption was investigated. The findings in Table 3 demonstrate that after being composited with graphene at 5000 rpm, BI’s specific surface area and pore volume increased, demonstrating the favourable impact of graphene on enhancing the specific surface area and pore volume of the nanocomposite. These results indicate the increase of oil absorption ability and better separation of oil from water by the prepared nanocomposite.

**Table 3:** BET Results.

Materials	Pore volume (m^3^/g)	Pore size (Å)	Surface area (m^2^/g)
BIG	0.0178	128.0	5.55
BI	0.0077	80.9	3.82
IP	0.0013	101.4	0.50
RI	0.0175	95.1	7.37
YI	0.0366	78.2	18.68

#### Oil absorption test by graphene and iron oxides nanocomposites

**Figure 13:**
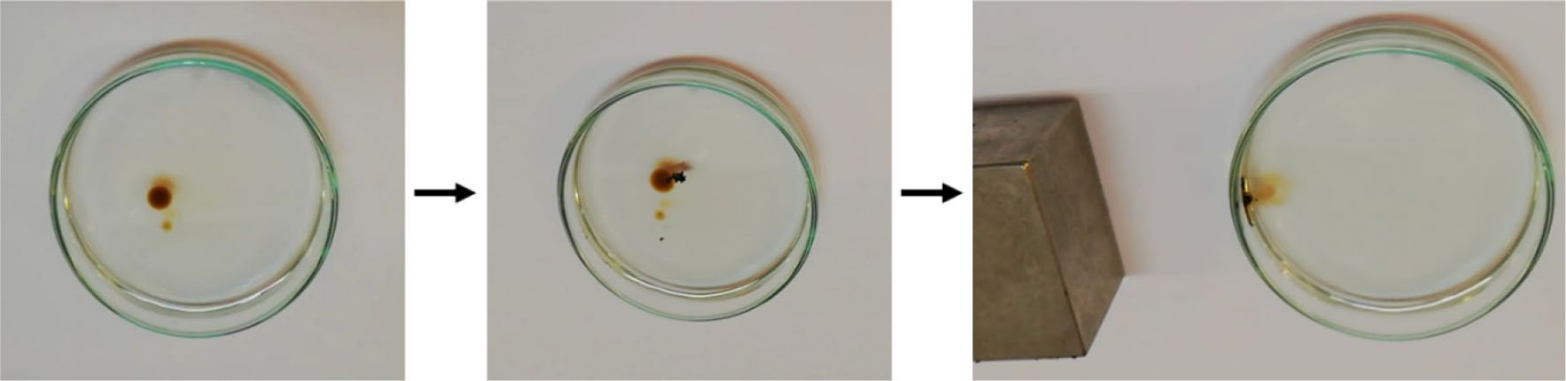
The photo of separating oil from water with BIG nanocomposite (5000 rpm) by magnet.

After pouring the BIG nanocomposite powder on the beaker surface containing oil and water, the oil on the water surface was absorbed by the nanocomposite powder. Then, by bringing the magnet closer, the rate of oil absorption by the nanocomposite increased so that the oil was completely absorbed by the nanocomposite (Fig. 13). Video 1 demonstrates how the oil-containing nanocomposite flows towards the magnet, where it is readily separated from the water by the magnetic field produced by the magnet and sticks to the beaker’s upper wall. The same procedure was performed for all prepared nanocomposites. The absorption of oil was observed for the BIG nanocomposite with graphene at 5000 rpm as optimized sample in the presence of the magnetic field (Fig. 13). For determining the oil absorption capacity of BIG, powder and oil were mixed in a ratio of 1–8, then by the magnet, the powder and absorbed oil were separated and the non-adsorbed oil was decanted. The absorption capacity of BIG was determined according to Eq. (1) which m_0_ is the powder weight before oil adsorption and m_1_ is the powder weight after oil absorption. The absorption capacity was obtained 2.8 g g^−1^.

1 $$Q = \frac{{\left( {m_{1} - m_{0} } \right)}}{{m_{0} }}$$

## Conclusion

Graphene nanocomposites/iron powder and graphene nanocomposites/black, red and yellow iron oxides were prepared. The contact angle data showed that these nanocomposites are highly-hydrophobic and some of them are smart with respect to the magnetic field. Among these nanocomposites, the nanocomposite prepared with 3000 rpm graphene and RI showed the highest response to the magnetic field. So that by applying the magnetic field, the nanocomposite becomes more hydrophobic and absorbs more oil, and the oil is separated from it after removing the field. The prepared nanocomposites can absorb oil both when they are placed on the substrate and when they are used in the form of powder (without the need for a substrate). For oil absorption by the nanocomposites prepared in this work, unlike other methods that have been presented so far, no substrate is needed, and as a result, both time and money are saved. Nanocomposites are made from environmentally friendly materials and do not pollute the environment. Easily and with the help of magnetic field (magnet), nanocomposite powder can be separated from the water after absorbing oil. These nanocomposites are smart and most of them become more hydrophobic in the presence of a magnet and thus have a higher ability to absorb the oil. The magnetic field applied by a magnet to these nanocomposites, in addition to increasing the hydrophobicity and as a result of more absorption of oil by the nanocomposite, causes their complete separation from water. This study is ongoing to enhance absorption capacity while employing a straightforward approach for large-scale implementation in industries. Additionally, a combination of various iron oxides is being employed to achieve improved outcomes.

### Supplementary Information

Supplementary Information.

## Data Availability

The data underlying Figs. 2, 3, 4, 5, 6, 8, 10, 11, 12 and 13 and Table 3, Supplementary Figures S1–S5, and Table S1 are available from the corresponding author upon reasonable request.

## Abbreviations

IP: Iron powder
BI: Black iron oxide
RI: Red iron oxide
YI: Yellow iron oxide
IPG: Graphene@Iron Powder
BIG: Graphene@Black Iron oxide
RIG: Graphene@Red Iron oxide
YIG: Graphene@Yellow Iron oxide
FT-IR: Fourier transform infrared spectroscopy
EDS: X-ray energy dispersive spectroscopy
SEM: Scanning electron microscopy
VSM: Vibrating sample magnetometer
MSH: Magnetic smart hydrophobicity
