# Supplementary material for: Preparation of the graphene-based smart hydrophobic nanocomposite and its application in oil/water separation
Source: Sci Rep. 2023 Nov 13;13:19816. doi: 10.1038/s41598-023-46520-2 (PMC10643443; doi:10.1038/s41598-023-46520-2)
Supplement: Supplementary file 1 — Supplementary Information. [file 41598_2023_46520_MOESM1_ESM.doc]

**Supporting Information of**

**Preparation of the graphene-based smart hydrophobic nanocomposite and its application in oil/water separation**

Mahsa Alimohammadian^†^, Saeid Azizian^‡^, Beheshteh Sohrabi^†, *^

† Surface Chemistry Research Laboratory, Faculty of Chemistry, Iran University of Science and Technology, Tehran, Iran.

‡ Department of Physical Chemistry, Faculty of Chemistry, Bu-Ali Sina University, Hamedan, Iran.

*Corresponding author. Tel: +982173228315 Fax: +982177491204. E-mail: [Sohrabi_b@iust.ac.ir](mailto:Sohrabi_b@iust.ac.ir)

, [Sohrabi_b@yahoo.com](mailto:Sohrabi_b@yahoo.com) (Beheshteh Sohrabi)/


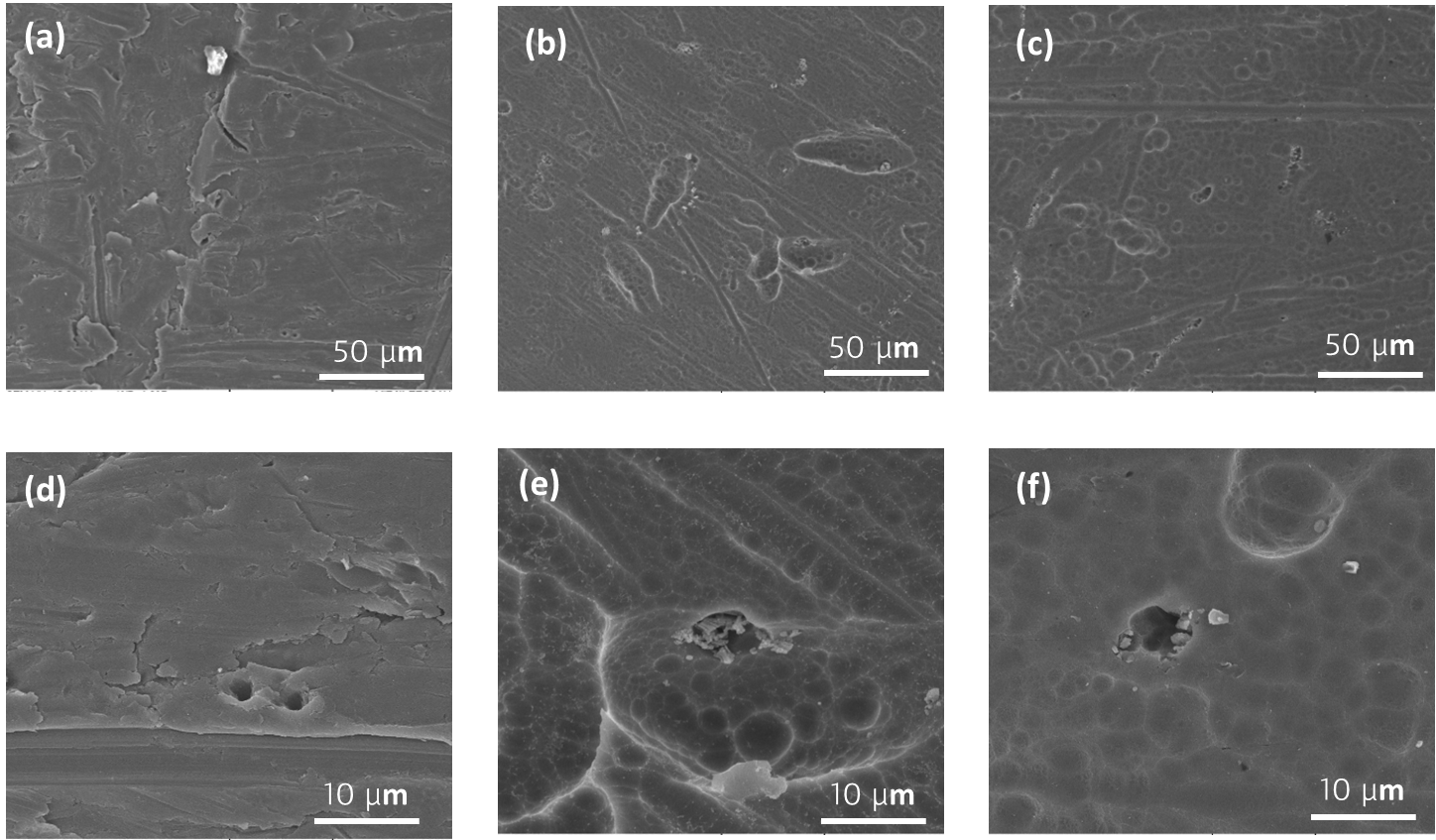


**Figure S1**. The FESEM images of aluminum alloy surfaces with different magnifications.

a, d) Aluminum alloy before engraving. b, e) aluminum alloy etched by alkaline method. c, f) Aluminum alloy etched by chemical method.


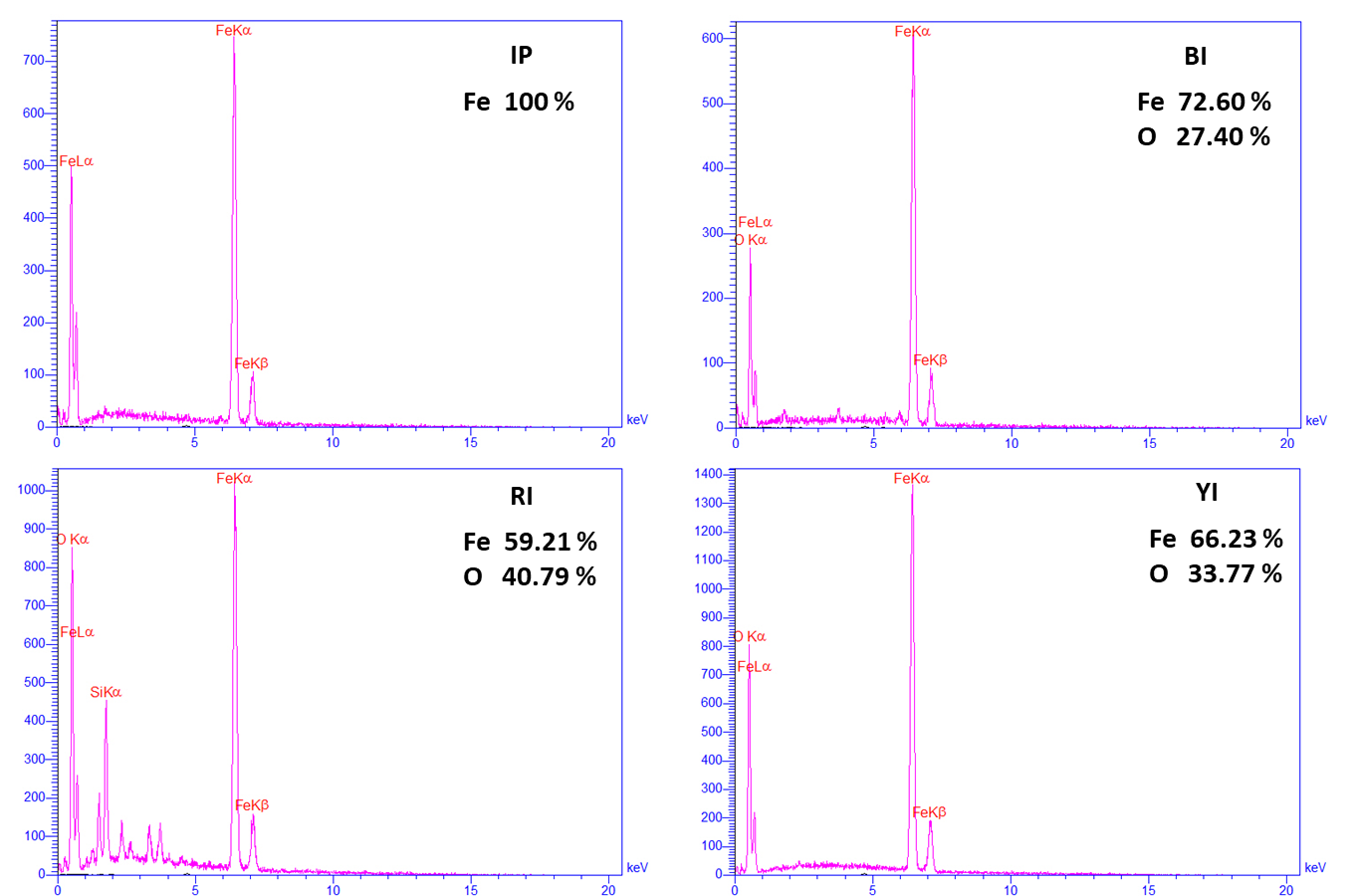


**Figure S2**. EDS analysis pristine IP, BI, RI and YI.


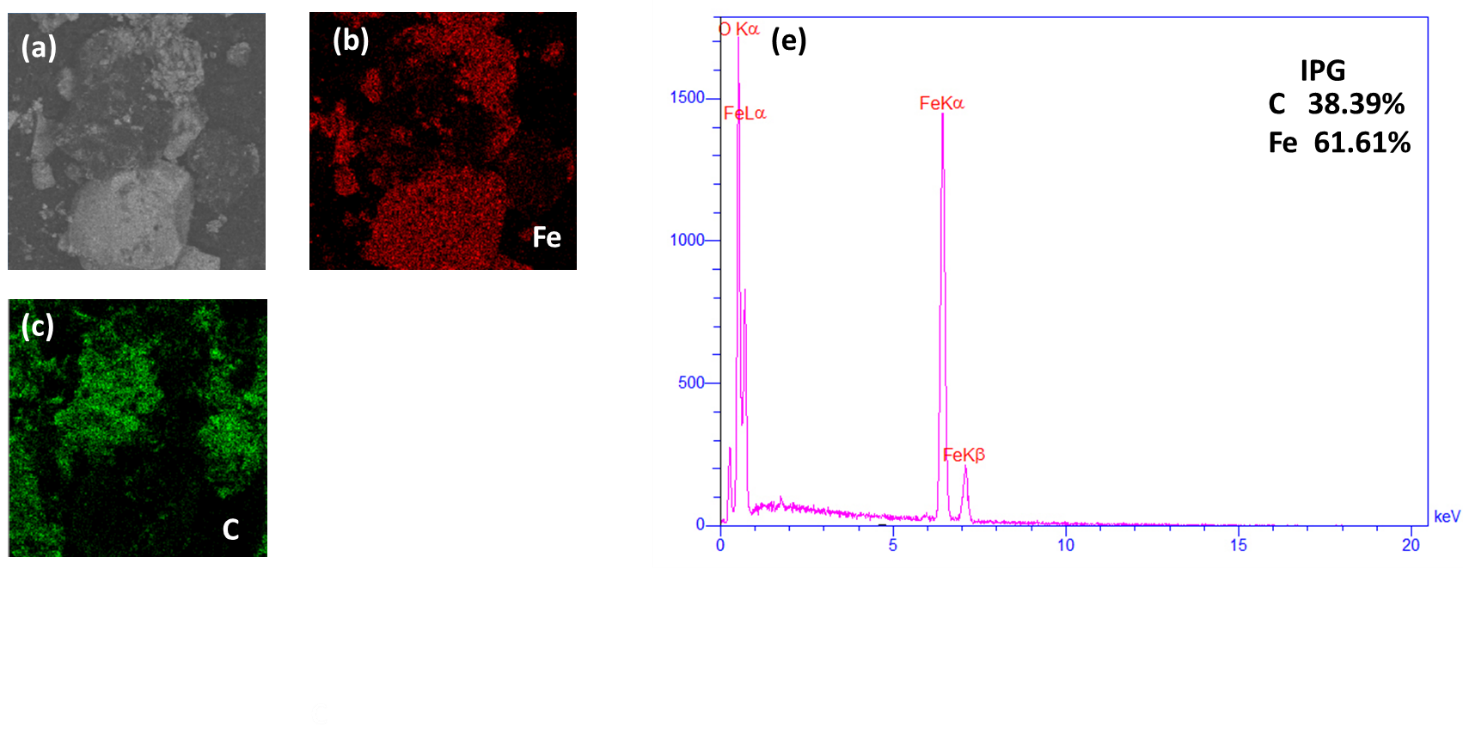


**Figure S3**. Map and EDS analysis for iron powder graphene nanocomposite: (a) SEM image (b) Fe map (c) C map (e) EDS spectrum.


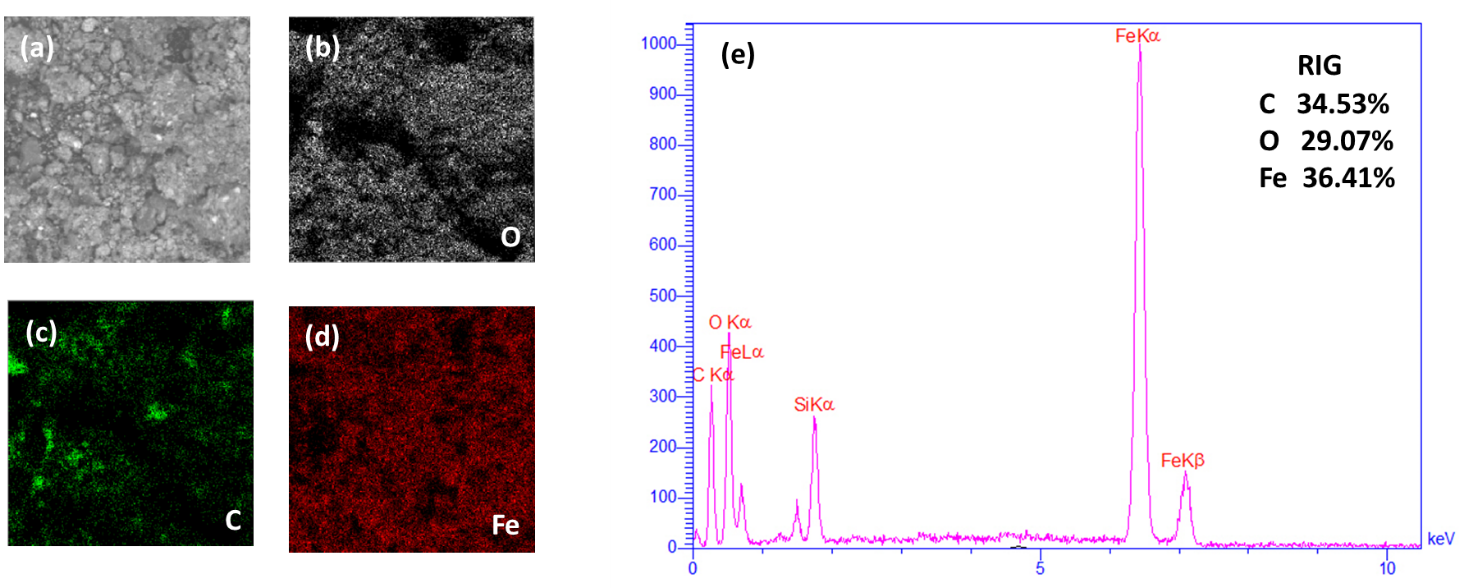


**Figure S4**. Map and EDS analysis for red iron oxide graphene nanocomposite: (a) SEM image (b) O map (c) C map carbon (d) Fe map (e) EDS spectrum.


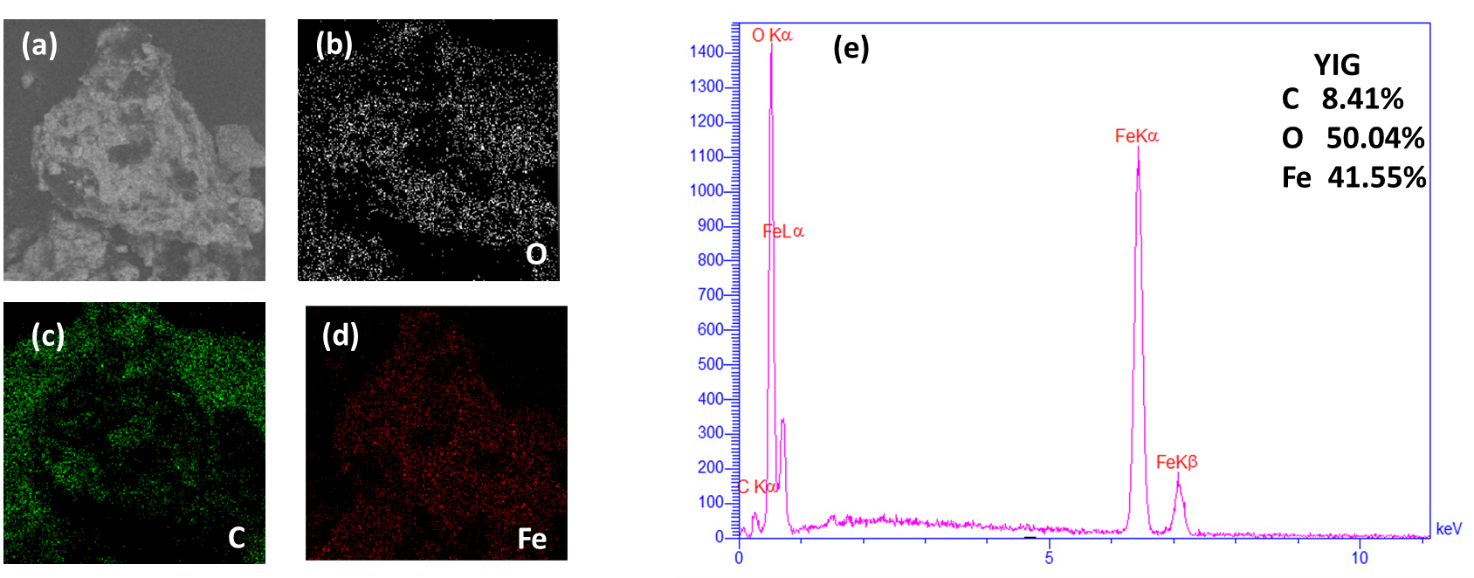


**Figure S5**. Map and EDS analysis for yellow iron oxide graphene nanocomposite: (a) SEM image (b) O map (c) C map (d) Fe map (e) EDS spectrum.

| Table S1. Saturation magnetization (M_S_) for all samples | |
| --- | --- |
| **Sample** | **M_S_ (emu/g)** |
| Pure Iron powder | 44.8 |
| Black Iron Oxide | 24.8 |
| Red Iron Oxide | 0.59 |
| Yellow Iron Oxide | 0.49 |
| Black Iron Oxide Graphene nanocomposite | 36.2 |
| Graphene 3000 rpm | 0.11 |
| Graphene 5000 rpm | 0.33 |
